# Which Role Plays 2-Hydroxybutyric Acid on Insulin Resistance?

**DOI:** 10.3390/metabo11120835

**Published:** 2021-12-03

**Authors:** André P. Sousa, Diogo M. Cunha, Carolina Franco, Catarina Teixeira, Frantz Gojon, Pilar Baylina, Ruben Fernandes

**Affiliations:** 1 Laboratory of Medical & Industrial Biotechnology (LABMI), Porto Research, Technology & Innovation Center (PORTIC), R. Arquitecto Lobão Vital 172, 4200-374 Porto, Portugal; andre.mp.sousa2@hotmail.com (A.P.S.); catarinateixeira126@gmail.com (C.T.); frantz.estsp@gmail.com (F.G.); pilarbaylina@ess.ipp.pt (P.B.); 2 School of Health (ESS), Polytechnic Institute of Porto (IPP), R. António Bernardino de Almeida 400, 4200-072 Porto, Portugal; diogofrancisco_07@hotmail.com (D.M.C.); 10190673@ess.ipp.pt (C.F.); 3 Faculty of Medicine, Porto University (FMUP), Alameda Hernâni Monteiro, 4200-319 Porto, Portugal

**Keywords:** 2-hydroxybutyric acid, impaired glucose tolerance, insulin resistance, type 2 diabetes mellitus

## Abstract

Type 2 Diabetes Mellitus (T2D) is defined as a chronic condition caused by beta cell loss and/or dysfunction and insulin resistance (IR). The discovering of novel biomarkers capable of identifying T2D and other metabolic disorders associated with IR in a timely and accurate way is critical. In this review, 2-hydroxybutyric acid (2HB) is presented as that upheaval biomarker with an unexplored potential ahead. Due to the activation of other metabolic pathways during IR, 2HB is synthesized as a coproduct of protein metabolism, being the progression of IR intrinsically related to the increasing of 2HB levels. Hence, the focus of this review will be on the 2HB metabolite and its involvement in glucose homeostasis. A literature review was conducted, which comprised an examination of publications from different databases that had been published over the previous ten years. A total of 19 articles fulfilled the intended set of criteria. The use of 2HB as an early indicator of IR was separated into subjects based on the number of analytes examined simultaneously. In terms of the association between 2HB and IR, it has been established that increasing 2HB levels can predict the development of IR. Thus, 2HB has demonstrated considerable promise as a clinical monitoring molecule, not only as an IR biomarker, but also for disease follow-up throughout IR treatment.

## 1. Introduction

Diabetes mellitus type 2 (T2D), commonly known as non-insulin-dependent diabetes mellitus (NIDDM) is responsible for up to 95% of diabetic cases worldwide [1]. It is defined as a chronic condition characterized by the loss and/or dysfunction of β-cells and insulin resistance (IR) in effector tissues, which is immediately recognized by an increase in glucose levels in the bloodstream, i.e., hyperglycemia [2,3,4].

The prevalence of T2D is increasing globally owing to population aging, the predominance of sedentary lifestyles in major western cultures and economic differences between developed and developing countries. According to the International Diabetes Federation’s most recent reports, the number of undiagnosed people with diabetes has reached concerning levels, with 232 million people remaining undiagnosed for diabetes, indicating a dire need for new diagnosis methods and techniques that are both quick and inexpensive. The diabetic population is anticipated to reach 578 million people by 2030 and 700 million by 2045, with an increase in the death rate. Furthermore, 374 million people are at risk or extremely at risk of developing T2D, mostly in developed countries.

Only in the United States in 2019, at least USD 760 billion was spent on public health for diabetes prognosis and treatment. It becomes clear what future challenges governments and associations will face in order to change this reality and find answers to a growing problem and disease in the industrialized world [1].

Diabetes has already been associated with a high death rate; however it frequently leads to more serious comorbidities such as cardiovascular diseases (CVD), heart strokes, neuropathies, nephropathies, retinopathies, pulmonary diseases, depression, dementia, cancer, infectious diseases and, ultimately, to death [2,5].

### 1.1. Insulin, a Powerful Molecule

Insulin, a peptide hormone with a molecular weight of 5808 kDa, is composed of 51 amino acids distributed in an α and β chain joined by two disulfide bridges. It belongs to a family that includes insulin-like growth factors (IGF) I and II, relaxin, and other insulin-like peptides that allow maintaining glucose homeostasis. Insulin is synthesized in pancreatic islet β cells; however, its precursor is known as preproinsulin. This peptide is converted to proinsulin by microsomal enzymes, cleaves the N-terminal signal peptide and stores it in zinc-bound crystals where proteases can work, resulting in insulin maturation and exocytosis in response to an appropriate stimulus [6,7].

Insulin’s main role in the metabolism is to promote the entry of glucose into the cells to be used as a substrate for glycolysis, generating energy reserves. Additionally, insulin is involved in processes closely related to the central nervous system (CNS) and the cardiovascular system (CVS), cellular proliferation and growth, as well as with the regulation of glucagon (potent inducer of hyperglycemia) secretion from pancreatic α-cells [8,9].

### 1.2. Insulin Resistance

T2D is a metabolic condition that impairs glucose metabolism while increasing the activity of other anabolic and catabolic pathways. Most cells use glucose as substrate, which requires insulin signaling. IR is a common occurrence in the development of T2D and occurs when cell receptors lose their capacity to recognize and connect with insulin, resulting in the failure of vital molecular pathways to the cells. Aside from being a risk factor for T2D, it is also linked to metabolic syndromes, obesity, dyslipidemia, hypertension, atherosclerosis, non-alcoholic fatty liver disease, among others [2,10,11].

Glucose transport (GLUT) across the plasma membrane is performed without the use of energy in favor of glucose gradient through the GLUT transporters. These are linked to the hexokinase enzyme, which initiates intracellular signaling by promoting glucose phosphorylation to glucose 6-phosphate (G6P). In this situation, because it is dependent on insulin as a beginning signal, GLUT-4 is the most affected receptor by IR [11].

Insulin signaling pathways are activated when insulin binds to the tyrosine-kinase domain of the transmembrane receptor. Endogenous ligands then bind to the domain, causing auto-phosphorylation in the tyrosine residues. This is followed by downstream events such as recruitment of the adaptor proteins insulin receptor substrates (IRSs) and several other adapter proteins, which creates a binding site for IRS-1 that phosphorylates by insulin-induced kinases. Activated IRS-1 interacts with phosphoinositide 3-kinase (PI3K), activating the complex and catalyzing the conversion of phosphatidylinositol 4,5-bisphosphate (PIP2) to phosphatidylinositol 3,4,5-trisphosphate (PIP3). PIP3 is a powerful inducer of protein kinase activity, particularly protein kinase B (PKB or Akt). PKB promotes glucose entry into cells by translocating GLUT-4 to the membrane and inhibiting proteins responsible for promoting glycogen synthesis. Thus, a disruption in the signaling cascade or the blockage of receptors will result in a hyperglycemic state in which the cells are unable to take in glucose [8,12,13,14].

### 1.3. Mechanisms Underlying the IR Effect

Several mechanisms, as previously documented in the literature, highlight the IR effect. Ref. [15] identified protein-tyrosine phosphatase 1b (PTP1B) as a negative modulator of insulin signaling due to a reduction of insulin-induced phosphorylation of IRS-1 tyrosine residues and, as result, inhibits residual signal transduction.

Several cytokines and inflammatory mediators are upregulated in IR, according to [16,17] and others, including tumor necrosis factor-α (TNF-α), monocyte chemotactic protein-1 (MCP-1), C-reactive protein (CRP), and interleukins. TNF-α supresses GLUT-4 expression by serine phosphorylating IRS-1, hence inhibiting insulin cascade phosphorylation. According to [18], the involvement of IKKβ/NF-Κb and JNK pathways in IR increases, since a main result of the JNK pathway is the serine phosphorylation in IRS-1, impairing insulin signaling, and as a result of the activation of IKKβ/NF-Κb pathway in adipocytes and skeletal muscle, IL-1 reduces IRS-1 expression via the ERK1/2 pathway. Ref. [19] reported that IL-6 induces ubiquitylation and [18] reported that inflammation-induced nitric oxide production suppresses the PI3K–Akt pathway.

Ref. [20] observed that oxidative stress can promote IR via affecting insulin signal transduction, since oxidative stress can also activate IKKβ/NF-κB and JNK, which phosphorylates IRS and cause it to degrade. Ref. [21] also decrease GLUT-4 localisation in cell membranes.

According to [22], any abnormality in the serine phosphorylation of IRS-1 can affect insulin signal transduction. Refs. [23,24] report that defect can be due to a reduction in IRS-1 phosphorylation or an increase in IRS-1 phosphorylation at serine 307, which inhibits insulin signal transduction and leads to IR.

Finally, the protein affected by insulin signaling pathway disruption is the transporter GLUT-4, which plays a critical role in the cell’s glucose intake. A mutation in the transporter, on the other hand, could alter its normal function and prevent glucose transportation. Stress in the endoplasmatic reticulum (ER) can also lead to the development of IR. The interruption of normal ER function has an effect on the pancreatic β-cells responsible for insulin biosynthesis [25].

Since T2D is so closely related to IR, it is critical to establish biomarkers that allow recognizing these pre-diabetic states, so actions can be taken sooner [2,3,26]. To be considered as a novel biomarker, the compound must be easily measurable using different methodologies and techniques, capable of shortening the diagnosis timeline, and capable of preserving the proteins and components of the sample. This would enable for a significantly more precise and earlier diagnosis, as well as tracking illness progression and the therapeutic response while also studying the subjacent mechanisms of IR [27,28].

Through a metabolomic approach, several compounds were associated with IR including BCAA (branch chain amino acids)—leucine, isoleucine, valine–serine, 2-hydroxybutyric acid (2HB), β-hydroxybutyrate (β-HB), glycerylphosphorylcholine (L-GPC), inositol, oleic acid, D-glucose, 2-aminobutyric acid, 2-hydroxyvaleric acid and γ-glutamyleucine. These metabolites are linked to pancreatic β-cells dysfunction and T2D.

Nonetheless, this review will focus on the metabolite 2HB since it has been demonstrated that this compound can be detected and even measured in samples from different sources (saliva, urine, sweat, others) [26,27,29]. High serum levels of 2HB indicate disrupted homeostasis of the insulin–glucose relationship, despite the levels varying depending on gender, age, and body mass index [2,26,28]. It has also been demonstrated that targeting this biomarker could improve the therapeutic response for IR [30].

2-Hydroxybutyric acid (2HB) is an organic acid that has a carboxylic acid and is substituted with a hydroxyl group on the adjacent carbon. It is a chiral compound with two enantiomers, D-2-hydroxybutyric acid and L-2-hydroxybutyric acid, both of which are fully functioning molecules. 2HB is a by-product of the synthesis of 2ketobutyrate (2KB) which is mediated by lactate dehydrogenase (LDH) or 2hydroxybutyrate dehydrogenase (2HBDH), a type of LDH found in the heart. These enzymes’ activity is linked to a high dihydronicotinamide adenine dinucleotide/nicotinamide adenine dinucleotide (NADH/NAD^+^) ratio [31,32]. It has been identified as a persistent biomarker associated with insulin sensitivity, T2D and major cardiovascular illness, which is involved in lipid oxidation and oxidative stress [4].

### 1.4. Biosynthesis of 2HB

As a by-product of glutathione anabolism (cysteine synthesis pathway) and amino acid catabolism (threonine and methionine), 2KB is generated. It is promptly degraded to carbon dioxide and propionyl-CoA, an essential substrate in the citric acid cycle, which provides energy in form of ATP to the organism [31].

In an initial stage, there are two key pathways that converge into 2KB: (1) is the threonine and methionine catabolism, responsible for producing 2KB to enter the propionate catabolic pathway towards succinyl-CoA. Homocysteine is linked by serine in the methionine catabolic pathway to generate cystathionine, which is converted into cysteine, 2KB and ammonia by cystathionine γ-lyase [13]. In the threonine catabolic pathway, the amino acid is converted in 2KB by threonine dehydratase enzyme, resulting in the formation of H_2_O and ammonia [33]; (2) the synthesis of glutathione as a result of increased oxidative stress. This type of stress causes the transsulfuration of homocysteine to produce cystathionine and consequently the production of cysteine for glutathione synthesis, culminating in 2KB as an intermediate product [34].

The conversion of 2KB to the 2HB belongs to a later stage. This outcome is expected if the rate of 2KB synthesis exceeds the rate of catabolism, resulting in 2HB production or inhibition of the dehydrogenase that catalyzes the conversion of 2KB to propionyl-CoA [31,32].

### 1.5. HB Pathways Associated with IR

Overall, 2HB concentration is strongly linked to impairment of β-cells function in the human body and an increase in free fatty acids (FFA) in circulation, as well as to increased oxidative stress, which is a hallmark of an IR state [2].

**Figure 1:**
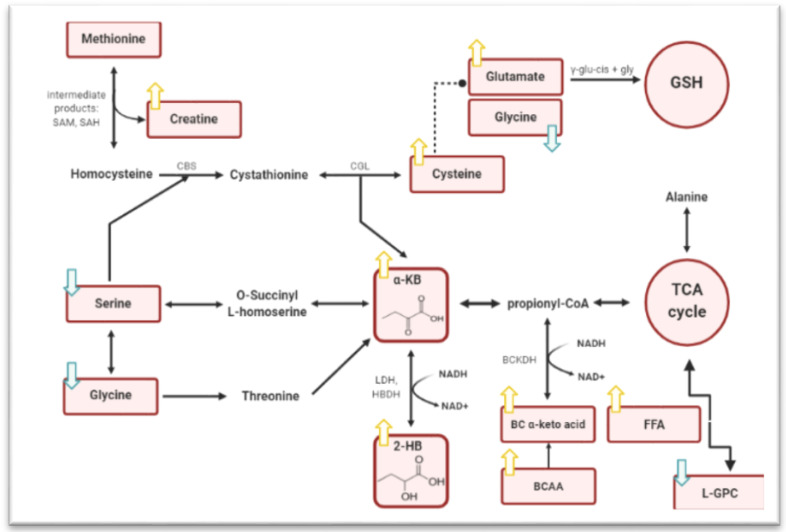
Model of the biochemical relationship of 2HB biosynthesis and associated metabolic pathways with IR. 2HB is produced from the conversion of α-KB in a reaction catalyzed by LDH that occurs when the NADH/NAD+ ratio is elevated, as can occur from higher lipid oxidation events. 2HB–2-hydroxybutyrate; 2KB (α-KB)–alpha-ketobutyrate; BCAA, branched chain amino acids; BCKDH–branched chain alpha keto acid dehydrogenase; CBS–cystathionine-beta-synthase; CGL–cystathionine gamma-lyase; HBDH–α-hydroxybutyrate dehydrogenase; LDH–lactate dehydrogenase; SAH–S-adenosyl-L-homocysteine; SAM–S-adenosyl Methionine; FFA–Free Fatty Acids; L-GPC–glycerylphosphorylcholine.

Under hyperglycemic circumstances caused by IR, more glucose will flow through the glycolytic pathway, producing more pyruvate and acetyl-CoA, leading to more NADH synthesis. This way, NADH accumulates and initiates an electron pressure on the mitochondrial electron transport chain, resulting in oxidative stress since NAD^+^ is not supplied. Despite the fact that glutathione (GSH) exhibits antioxidant properties and is useful to balance the system when NADPH levels become lower, the GSH is unable to regenerate. As a result, cellular antioxidant activity may be compromised, resulting in increased levels of reactive oxygen species capable of attacking macromolecules and inducing oxidative damage. To overcome the oxidative stress, hepatic cells generate GSH by cysteine anabolism, which produces the by-product α-ketobutyrate that will be then converted to 2-hydroxybutiric acid. Simultaneously, FFA plays a key role and is inextricably linked to IR. Lipid oxidation (triglycerides and phospholipids are hydrolyzed by cellular lipases) in IR-treated adipose tissue in increased FFA concentrations, which are oxidized by the TCA cycle and hence produce NADH [35]. Such a TCA cycle excess results in the accumulation of amino acids such as glutamate and alanine, as well as αKB, the precursor for 2HB [27,36]. It is worth noting the symbiotic effect between the FFA entrance in non-adipose tissues and the development of IR [35]. In the end, 2HB levels and biological activity are highly translated in the human body (Figure 1).

### 1.6. A Valuable Biomarker

Treating 2HB as an early indicator for T2D has obtained widespread acceptance in the scientific community throughout the years. Although it is controversial since it is present in several biochemical pathways, its action in lactic acidosis and ketoacidosis, as well as in the plasma of healthy subjects during prolonged fasting, indicates a role in a disordered metabolism and, as a result, a possible biomarker for T2D [36,37]. As stated in this review, 2HB is fundamentally related to insulin resistance and from excessive fat and protein intake, which leads to impaired insulin signaling and mitochondrial overload, which are the key characteristics of T2D and the cause of its complications and symptomatology.

### 1.7. Quantification of 2HB

2HB has been previously identified using three different methods: LC-MS [10,38,39,40], GC-MS [10,38,40,41] and NMR [38,40]. The samples were derived from several biospecimens.

The compound was detected and quantified in blood with a normalized value ranging from 8.00–80.0 µM [42], in cerebrospinal fluid (CSF) with a normalized value of approximately 37.0 ± 24.0 µM depending on the study [40,42,43], in saliva with a normalized value of 10.42 ± 9.20 µM [44], and in urine, although the values differ greatly from study to study and so, there is not a normalized and consensus value [31,38,45,46]. The compound was also found in feces [47,48] and sweat [49], but was not quantified.

Even though 2HB is a novel biomarker for T2D and has the potential to be a key differentiator for prevention, early diagnosis, and treatment, it occurs with limitations. As a result, it is required to develop a process that can improve a more precocious and certain diagnosis. Owing to advancements in metabolomics, it is now possible to uncover biomarkers that can provide insights into complex metabolic illnesses, as well as monitor and predict responses to therapeutic interventions, all with a single fasting plasma sample [10,36,50].

### 1.8. The Novel Quantification Method, Quantose M^Q^

Eventually, numerous metabolites altered in T2D, IR and IGT were researched and a connection was discovered, resulting in a test that correlates four variables: 2HB, oleate, insulin and L-GPC. Quantose M^Q^ is a test that can predict the progression from normal glucose tolerance to impaired glucose tolerance and, finally, diabetes [36]. Scores above 63 are indicative of IR [51].

Quantose M^Q^ and its metabolites serve as a foundation for research into IR and prediabetes since their alterations correlate strongly with changes in insulin sensitivity and glucose tolerance status. This novel fasting plasma measurement provides a turning point as a viable approach to illness diagnosis and progression evaluation [52].

Nevertheless, it is critical to identify biomarkers and clinical tests/methods that are both user-friendly and low-cost in order to withstand and prevent the development of T2D in its early stages.

Thus, the goal of this review is to unravel the potential of 2HB as an IR predictor independently or associated with other biomolecules, as well as the current methodologies available for detecting it.

## 2. Results and Discussion

In order to verify the potential of 2HB as an appropriate biomarker to detect early IR, a compilation of the outcomes is presented in Table 1 that will be explored below.

### 2.1. 2HB

Ref. [26] has looked through the main biofunction of 2HB and his results pointed that this biomolecule is synthesized in response to the oxidative stress and lipid peroxidation caused by IR and IGT regulation; in this way, it is considered as an early marker to both conditions. This author has also settled a 2HB concentration cut-off of 5 µg/mL, with values above indicating the presence of IR and IGT. Furthermore, [29], in his study, demonstrated that high levels of 2HB are common in T2D, also suggesting that it can be caused by the IR. Furthermore, Varvel, S. (2015) showed that measuring serum 2HB is a reliable method to screen hyperglycemia and β-cell dysfunction, giving fast results without using many resources. Salgado-Bustamante, Ref. [56] reported that an increase of 2HB was reported in the urine of T2D patients.

Looking specifically at IR and 2HB, Ref. [57] has demonstrated that 2HB levels increased in IR, potentially due to metabolic overload (through BCAA and free fatty acids) and oxidative stress (by the higher intracellular NADH/NAD^+^ ratio). In addition, the author showed that the pharmacological approach against IR resulted in a decrease in 2HB levels, which is consistent with the results presented until now.

The mechanism adjacent to IGT was also explored in order to better understand the biopathology underlies IR. For that, Ref. [53] investigated the infection with *S. aureus* and reported that the bacteria produce an insulin-binding protein that reduces glucose uptake by blocking insulin receptors, resulting in metabolic syndrome. However, an antibody fully capable of disabling the blocker protein was developed, and 2HB was used to grant the effectiveness of the treatment. Results showed that levels of 2HB reduced among time of the study.

Ref. [56] verified the relation between low birthweight and IR, attending to 2HB levels. Even though results were only expressed in women, they showed that biochemical pathways modified on adulthood were a consequence of weight at birth, which are connected directly with IR.

In a different study, Ref. [30] investigated the effect of a gastric bypass surgery in the IR. He verifies that 2HB levels have decreased 6 months following surgery, when compared to the levels at the moment of the gastric procedure, which was an indicator of improvement of IR. The author suggested that this mechanism is due to a precursor of 2KB—2-ketobutyric acid—that is increased in oxidative stress conditions related to glutathione synthesis. When overexpressed, this metabolic pathway produces 2HB as a byproduct, which can be used to detect IR, e.g., being an early biomarker in the non-diabetic population [28].

In a study involving anticancer therapy for prostate cancer, Ref. [54] (2012) explored the effect of therapy on gonadal androgen therapy in IR. The results showed that 2HB levels decreased after 3 months of treatment.

Thus, the authors showed that higher levels of 2HB are an indicator of IR. It is important to refer to the fact that age, gender and body mass index do not interfere with any result.

### 2.2. Relationship between 2HB and Other Metabolites

Ref. [58] has defined the 2HB concentration in normal conditions as 1.60 ± 0.57 µg/mL. It was also described that α-tocopherol is dependent on 2HB to be synthesized. This way, an increase in 2HB leads to an increase in and α-tocopherol levels, which will on the one hand induce IR and IGT, and on the other hand increase other risk factors to T2D and/or hypertension development. Ref. [27] reported that high levels of 2HB and lower levels of L-GPC were associated with IR and IGT. These two markers were also used to control the progression of IR among 3 years, demonstrating that they are a good method to predict IR. During patients’ follow up, a reduction from 4.21 ± 2.01 to 3.83 ± 1.73 µg/mL in 2HB concentration and an increase from 15.41 ± 6.60 to 16.24 ± 7.03 µg/mL in L-GPC concentration. The author also showed that there is an inverse relationship between β-cell dysfunction and 2HB. All those factors lead to an increased risk of developing T2D. Ref. [30] has also reported that the increase of 2HB leads to a consequent reduction of L-GPC when IR is under treatment. Other study conducted by [59] showed the relationship between 2HB and Branched-Chain Amino Acids (BCAA), where the 2HB participates in substrate BCAA synthesis. They demonstrated that fasting concentrations of BCAA and 2HB can predict IR in youth, which can prevent risks of developing other diseases in adulthood. Furthermore, the author reported that these two markers can predict incipient deterioration of β-cell function and IGT. In a new study, Ref. [4] has reported that the use of 2HB as a biomarker accurately differentiates sensitive patients from those resistant to insulin. Along with 2HB, other molecules were described to improve the diagnosis. It was reported the enhanced YKL-40 and soluble CD36 released by IGF-1 activation, which lead to high levels of oxidative stress. Il-18 and resistin were also demarked to become overexpressed in IR, in this way potentiating the previously described oxidative stress. Finally, RBP4 and chemerin were reported with 2HB, once these molecules are related to the insensitivity of GLUT-4, an important glucose transporter in muscle cells. Thus, these biomolecules panels were considered to be good biomarkers for IR and endothelial dysfunction in T2D patients. Specifically, 2HB was directly related to the influence of β-cell action on insulin levels, increasing glutathione production.

### 2.3. Quantose M^Q^

The Quantose M^Q^ score is the result of the measure of insulin, 2HB, linoleoyl-glycerophosphocholine, and oleate. This score allows detecting patients with IR in earlier stages.

Some studies done by [60] demonstrated that Quantose M^Q^ was an efficient diagnostic biomolecular panel to IR, with 51% of the population being detected accurately. It also has been shown to reduce the Quantose score when insulin sensitivity and glucose tolerance are improved. Ref. [52] reported that the use of an antidiabetic medicine (pioglitazone) reduced the concentration of 2HB by 6% and of insulin by 2%. The author also wrote that it “may serve as a useful clinical test to identify and monitor therapy in insulin-resistant patients”, and is a possible marker for monitoring therapeutic intervention.

Furthermore, Ref. [51] showed that the mean Quantose M^Q^ score was elevated in IR patients. The author explored the reason for that increasing in the score, pointing to non-alcoholic fatty liver disease as a possible cause, which involves NAFLD (involved in oxidative stress, including a set of molecules such as adipokines, chemokine and pro-inflammatory cytokines). It was also described that the presence of other diseases and the condition of IR lead to the increase in Quantose score.

It is important to point again that IR was not related to sex, age, weight, and body mass index.

## 3. Materials and Methods

The review was written according to PRISMA guidelines from [61].

### Database Research

Research was done in April 2020 in B-On (specifically in science direct), PubMed and Google Scholar, aiming to understand the broad association between 2HB and the mechanisms that lead to IR. For that reason, a study was designed based on the PICO (Population, Intervention, Comparison and Outcome) methodology according to the COCHRANE recommendations. Population: people at risk to develop insulin resistance. Intervention: detection of alpha-hydroxybutyrate. Comparison: levels of α-HB in insulin-resistant individuals vs. susceptible individuals. Outcome: insulin resistance. To execute the research, a combination of MeSH terms was used, such as “Insulin resistance (D007333)” and “Alpha-hydroxybutyrate (C031570)”. It is noteworthy that repeated articles were excluded.

As a result, from research with MeSH terms, 29 articles were identified, with 28 from PubMed and 1 from B-On (Science Direct). For these 29 articles, inclusion, and exclusion criteria (Table 2) were applied.

**Figure 2:**
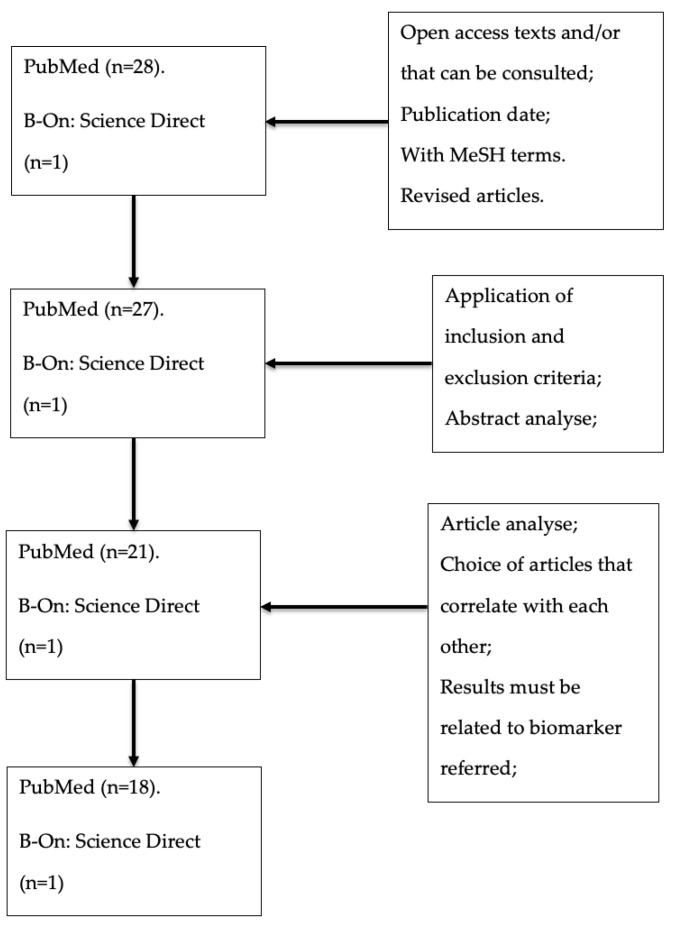
Study strategy to obtain the articles used in this review.

In addition, the abstracts of the articles were analyzed, as well as their discussion and results. Thus, several 22 articles were obtained—21 from PubMed and 1 from B-On. After verification and analysis of these 22 articles, only 19 fulfilled the other criteria present in Figure 2, 18 from PubMed and 1 from B-On.

Analysis of the analyte under study, associated diseases and methodology used for its analysis:

Table 1 represents the different results obtained in the articles analyzed, divided and 4 main dimensions: metabolite or group of metabolites studied; associated pathology/condition; applied methodology; outcomes.

## 4. Conclusions

New studies are needed to corroborate the relation between 2HB and IR, not only by in vitro studies that can validate this topic, but also by in vivo tests. Aside from its potential as an early biomarker for insulin resistance, 2HB has also demonstrated high potential to be a biomarker for tracking glycemic improvement, dysglycemia, hyperglycemia, β-cell dysfunction, lipid oxidation and oxidative stress, making it a biomarker for metabolic syndrome [28] Furthermore, it has an important role tracking the pathology‘s evolution, as shown by [30] who uses this biomolecule to track the evolution of IR after gastric bypass surgery. Moreover, in the studied literature, metabolomics approaches were a reliable method to detect and quantify 2HB.

Thus, according to the evidence included in this review, 2HB demonstrated considerable potential to be applied as an early biomarker for IR.

## Figures and Tables

**Table 1:** 2HB and related metabolites, pathologies and methodology of detection.

Study	Principal Metabolites Studied	Correlated Pathologic Conditions	Methodology	Outcomes
[29]	2HB	Insulin resistance (IR)	LC/MS and GC/MS	>levels of 2HB are related to diabetic and IGF patients
[28,30,53]			GC/MS	<2HB levels were observed after 6 months of gastric surgery. Furthermore, 2HB was used as inverse biomarker to predict improvement of pathology
[2]			HPLC and Oral Glucose Tolerance Testing (OGTT)	2HB can be used to predict hyperglycemia and β-cell dysfunction
[5]			LC/MS-MS and GC/MS	2HB showed in urine as a biomarker to T2D
[54]		IR and prostate cancer		2HB levels decreased after 3 months beginning treatment
[26]		IR and Impaired Glucose Tolerance (IGT)	UHPLC-MS/MS and GC/MS	2HB is an early marker for both IR and impaired glucose regulation
[55]			LC/MS-MS	Applied methodology was efficient to predict IR
[56]		IR and oxidative stress in low birthweight		Relation between low birthweight and IR
[57]		IR and dysregulations in thyroid hormone levels	UHPLC-MS/MS	2HB levels increased in IR, by metabolic overload and oxidative stress
[58]	2HB and α-tocopherol	IR and cardiovascular risk	UHPLC-MS and GC-MS	Increasing in 2HB and α-tocopherol levels were involved in IR and IGT
[3]	2HB and L-GPC	IR	n.m ***	High levels of 2HB and lower levels of L-GPC were associated with IR and IGT
[27]		IR and dysglicemia	HPLC-MS	
[59]	2HB and Branched-Chain Amino Acids (BCAA)	IR in youth	NMR and OGTT	BCAA and 2HB can predict IR in youth
[52]	Quantose M^Q^ mix *	IR and IGT	n.m ***	Improved insulin sensitivity and glucose tolerance, allowing to predict IR
[60]		IR and sclerosis multiple		Improved insulin sensitivity and glucose tolerance, allowing to predict IR
[51]		IR and non-alcoholic fatty liver disease and thrombocytopenia III	HPLC-MS and chemiluminescent microparticles immunoassay (for insulin specific)	Score was elevated in IR patients
[4]	Mix **	IR associated with atherosclerosis in coronary artery disease	n.m ***	A are a new set of biomarkers for IR and endothelial dysfunction in T2D patients
**Summary**	**2HB**	**Mainly IR**	**High throughput technologies (not routine methods)**	**Higher levels of** **2HB is positively associated with IR**

* α-hydroxybutyrate, oleate, insulin and L-linoleoyl-glycerophosphocholine (L-GPC). ** Mix—α-hydroxybutyrate, YKL-40, leptin, CD36, IL-18, RBP4, resistin, chemerin. *** n.m—not mentioned.

**Table 2:** Inclusion and exclusion criteria applied in the study.

Inclusion Criteria	Exclusion Criteria
Contain “abstract”; Be published and/or available to the public; Indicate the methodology used to detect 2HB; Indicate the relation between the metabolite and the mechanism of insulin resistance in pre-diabetic or T2D patients; Discuss relevant results; Highlight associated comorbidities that influences the measurement of analyte; Have been published between 2010 and the present; Language: English.	Do not demonstrate a direct effect of 2HB alone; Articles describing methods, models and theories without empirical data; Do not indicate the methodology used.

## Abbreviations

2HB/α-HB	2- or α-hydroxybutyric acid
2KB/α-KB	2- or α-ketobutyrate
3HB/β-HB	3- or β-hydroxybutyrate
BCAA	Branch chain amino acids
CNS	Central nervous system
CRP	C-reactive protein
CSF	Cerebrospinal fluid
CVD	Cardiovascular diseases
CVS	Cardiovascular system
ER	Endoplasmatic reticulum
FFA	Free fatty acids
G6P	Glucose 6-phosphate
GSH	Glutathione (reduced form)
HBDH	α-hydroxybutyrate dehydrogenase
WGS	Whole genome sequencing
XDR	Extensively drug-resistant
IGF	Insulin-like growth factors
IGT	Impaired Glucose Tolerance
IR	Insulin Resistance
IRS	Insulin receptor substrate
LDH	Lactate dehydrogenase
L-GPC	L-linoleoyl-glycerylphosphorylcholine
MCP-1	Monocyte chemotactic protein-1
NAD+	Nicotinamide adenine dinucleotide
NADH	Dihydronicotinamide adenine dinucleotide
NIDDM	Non-insulin-dependent diabetes mellitus
PI3K	Phosphoinositide 3-kinase
PICO	Population, Intervention, Comparison and Outcome
PIP2	y-linositol 4,5-bisphosphate
PIP3	Phosphatidylinositol 3,4,5-trisphosphate
PKB	Protein kinase B
PTP1B	Protein-tyrosine phosphatase 1b
T2D	Type 2 Diabetes Mellitus
TNF-α	Tumor necrosis factor-α
